# The tangled bank of amino acids

**DOI:** 10.1002/pro.2930

**Published:** 2016-05-12

**Authors:** Richard A. Goldstein, David D. Pollock

**Affiliations:** ^1^ Division of Infection and ImmunityUniversity College LondonLondonWC1E 6BTUK; ^2^ Department of Biochemistry and Molecular GeneticsUniversity of Colorado School of MedicineAuroraColorado80045

**Keywords:** protein evolution, molecular evolution, epistasis, epistatic interactions, substitution matrices, substitution rates, evolutionary process, phylogenetics, evolutionary Stokes shift

## Abstract

The use of amino acid substitution matrices to model protein evolution has yielded important insights into both the evolutionary process and the properties of specific protein families. In order to make these models tractable, standard substitution matrices represent the average results of the evolutionary process rather than the underlying molecular biophysics and population genetics, treating proteins as a set of independently evolving sites rather than as an integrated biomolecular entity. With advances in computing and the increasing availability of sequence data, we now have an opportunity to move beyond current substitution matrices to more interpretable mechanistic models with greater fidelity to the evolutionary process of mutation and selection and the holistic nature of the selective constraints. As part of this endeavour, we consider how epistatic interactions induce spatial and temporal rate heterogeneity, and demonstrate how these generally ignored factors can reconcile standard substitution rate matrices and the underlying biology, allowing us to better understand the meaning of these substitution rates. Using computational simulations of protein evolution, we can demonstrate the importance of both spatial and temporal heterogeneity in modelling protein evolution.

## Introduction

Darwin noted that the fitness of any organism is largely determined by its interactions with other organisms and the environments they produce, resulting in a ‘tangled bank’ of plants, birds, insects, and worms, all ‘dependent upon each other in so complex a manner’.^1^ This network of interactions extends the entire range of biologically relevant time and length scales, from the co‐evolutionary dynamics of hosts and their parasites, to selection for communal behaviour found in social insects, blind mole rats, and humans, to our dependence on the oxygen‐rich atmosphere formed by bacteria and plants.

When Perutz and Kendrew obtained the high‐resolution structure of myoglobin and haemoglobin, it became clear that the ‘tangled bank’ perspective is relevant for intra‐molecular interactions as well.^2^, ^3^ Proteins are structured, highly integrated entities. Natural selection acts on the ability of proteins to function and (for most proteins) to fold and be stable under physiological conditions. These properties are holistic, meaning that they are properties of the entire protein (or complex of proteins). Structure and stability depend on hydrogen bonds, ion pairs, packing interactions, and the formation of hydrophilic surfaces. Function involves forming high affinity binding sites and specific geometries of catalytic residues. The properties of the proteins are generated by the interactions between amino acids, so the selective constraints on a site in a protein can only be understood in the context of the amino acids forming the rest of the protein. A substitution at one site in a protein can affect the preferences for amino acids at other sites; the resulting substitutions at these other locations can then alter the selection at the first site, resulting in a complex network of feedback loops, a tangled bank of interactions. One aspect of this is the evolutionary ‘Stokes shift’, in which the rest of a protein adapts itself to a new amino acid resident at a given site, tending to make the resident amino acid more evolutionarily stable over time.^4^, ^5^

Because epistatic interactions result in great complexity, it is not surprising that they are strategically ignored in most phylogenetic modelling of protein evolution. This neglect has led to the production of powerful and computationally feasible approaches in which each site in a protein is assumed to evolve in a manner independent (and often identical) to evolution at other sites. These models can be adjusted to infer the numbers of different types of substitutions correctly, and thus reasonably well describe these *consequences* of epistatic interactions despite their independent‐site assumptions. The successes of such models came, however, at the cost of losing contact with the basic biological phenomena underlying the substitution process. For instance, models that incorporate the genetic code and represent some form of the process of mutation and selection can provide important insights about the selective forces acting on a protein inaccessible through purely empirical models.^6^, ^7^, ^8^ As computational methods advance and sequence data becomes more copious, new opportunities arise to go even further beyond empirical models, to connect with our underlying mechanistic understanding of protein biophysics and molecular evolution. At the same time, it is increasingly clear that epistatic interactions between sites in proteins can provide valuable information on protein evolution and the evolving proteins,^9^ and that it is perilous to ignore them.^10^

In this paper, we attempt to develop a unified framework to link the standard empirical and genetic code‐based models with the biophysical properties of proteins and the dynamics of evolution, and consider what these models can reveal about the underlying ‘tangled bank’ of amino acids in a protein. In particular, we consider the questions: What governs the relative rates of substitution? How do the substitution rates in empirical models relate to the underlying biophysics and evolutionary biology? Which aspects are well represented, missed, or in conflict with these standard models? How does the relative substitution rate between two amino acids depend on their physicochemical properties, including such factors as relative size, charge, and polarity? How is this dependence encoded in standard empirical and genetic code‐based models? And perhaps most importantly, how do both empirical and mechanistic approaches need to change to better represent the biological processes that they are attempting to model?

## Results

### Understanding relative substitution rates: Why substitutions are generally conservative

It has been observed many times that conservative mutations between similar amino acids are more likely to be accepted than mutations between dissimilar amino acids. This is observed, for instance, in the empirical substitution matrices created by Dayhoff and others.^11^, ^12^, ^13^, ^14^, ^15^ In such matrices, the substitution rate *Q*_IV_ between the aliphatic and hydrophobic amino acids isoleucine and valine is much higher than the rate *Q*_DL_ for the nonconservative substitution from negatively charged aspartic acid to neutral leucine. This can be interpreted as a simple consequence of an argument made by Fisher, who considered the focusing of a microscope; if the sample in the microscope is more or less in focus, turning the fine adjustment knob is much more likely to yield an improvement than turning the course adjustment knob.^16^ If you are near a fitness optimum, large steps (such as nonconservative amino acid changes) will tend to move you away from the optimum, rather than nearer to the peak, especially if the number of adjustment knobs is large. The physiochemical distance between amino acids is symmetric; both isoleucine to valine and valine to isoleucine substitutions are conservative and therefore expected to be fast, while both aspartic acid to leucine and leucine to aspartic acid substitutions are nonconservative and thus slow. This symmetry is encapsulated in the standard formation of these substitution models, where *Q*_XY_, the rate of substitution from amino acid *X* to *Y*, is represented as *Q*_XY_ = *S*_XY_ π_Y_, where π_Y_ is the observed equilibrium frequency of amino acid *Y* in the protein or protein database, and the exchangeability matrix *S*_XY_ is symmetric (e.g., *S*_XY _= *S*_YX_) with high values for pairs of similar amino acids and low values for dissimilar amino acids. This formulation guarantees ‘reversibility’, that is, at equilibrium the expected number of substitutions from *X* to *Y* (proportional to π_X_ *Q*_XY _= *S*_XY_ π_X_ π_Y_) is equal to the expected number of *Y* to *X* substitutions (proportional to π_Y_ *Q*_YX _= *S*_XY_ π_X_ π_Y_).^17^

In contrast to these standard empirical models of substitution, protein biophysicists and bioinformaticians often model how appropriate different amino acids are for a given site. This is, for instance, the principle behind hidden Markov models (HMMs) used to classify and cluster protein sequences;^18^, ^19^, ^20^ in these models, each site in the protein is represented as a node in a network characterised by ‘emission probabilities’ equal to the probability of observing a given amino acid at that site. Importantly, these probabilities are specific to each individual site, with local preferences for hydrophobic or helix forming, aliphatic, or specific amino acids depending upon structural and functional constraints. Recognition of distant homologues or proteins with similar structures depends on the site‐specific nature of these probabilities. This is in stark contrast to standard substitution models which generally assume that the same model applies at all sites in the protein at all points in evolutionary time (or include a site‐specific scaling factor that allows differences in average rates but does not affect relative rates^21^, ^22^).

Halpern and Bruno included this biophysical perspective in their evolutionary mutation‐selection models by incorporating the relative ‘fit’ of the wild type and mutant amino acid at any site, where amino acid fitness is similar to the concept of relative emission probabilities in an HMM.^23^ The mutation rate is multiplied by the probability of fixation to obtain the substitution rate; conservative changes tend to be close to neutral and accepted at rates similar to the neutral rate. Nonconservative substitutions are slower or faster than neutral substitutions depending upon whether they result in deleterious or advantageous changes in the protein. As a result, if a nonconservative change (e.g., from cysteine to proline) is highly deleterious with a low fixation probability resulting in a very low substitution rate, the opposite nonconservative change (e.g., from proline to cysteine) will be *advantageous* and thus have a substitution rate substantially higher than neutral, rather than lower as in standard substitution models. Mutation‐selection models have been used to estimate selection on degenerate codons,^24^ generate amino acid propensity profiles,^25^ characterize the distribution of fitness effects,^26^ and to identify changes in selective constraints following host shifts of pathogens.^8^, ^27^

Site heterogeneity is explicitly included in the mutation‐selection models, with the relative fitnesses of the amino acids defined for each site. This variation in relative fitness among sites leads to variation in relative rates of a form generally missing from standard empirical models, which must be considered if we wish to reconcile the standard substitution models with the biophysical description. Instead of rates, let us consider the flux Φ_*L*__,XY_ from amino acid *X* to *Y* at site *L*, that is, the number of times an *X* to *Y* substitution occurs at this site in a specified length of evolutionary time. The expected flux at this site is equal to $\Phi_{L,\text{XY}}=\pi_{L,X}Q_{L,\text{XY}}$, so the estimated rate constant is ${\hat{Q}}_{L,\text{XY}}=\frac{\Phi_{L,\text{XY}}}{\pi_{L,X}}$, where the site‐specific selective constraints are explicitly represented. Thus, the relative equilibrium frequencies and substitution rates also depend on the site in the protein. If we wish to develop a standard substitution model that ignores this site heterogeneity, we must average both observed substitutions and equilibrium frequencies over all of the sites, to generate an estimated rate given by ${\hat{Q}}_{\text{XY}}=\frac{{〈\Phi_{L,\text{XY}}〉}_{L}}{{〈\pi_{L,X}〉}_{L}}=\frac{{〈\pi_{L,\text{XY}}Q_{L,\text{XY}}〉}_{L}}{{〈\pi_{L,X}〉}_{L}}$.

This quantity can be calculated by modelling the evolutionary dynamics using the diffusion‐based formulation of Kimura.^28^ According to this formulation, the substitution rate from amino acid *X* to *Y* at site *L* is given by:

(1) $$Q_{L,\text{XY}}=v_{\text{XY}}\frac{4N_{e}(m_{L,Y}-m_{L,X})}{1-e^{-4N_{e}(m_{L,Y}-m_{L,X})}}$$

where $v_{\text{XY}}$ is the codon‐averaged relative mutation rate, $m_{X}$ is the marginal Malthusian fitness of an individual with amino acid *X* in site *L*, and *N*_e_ is the effective population size. The corresponding equilibrium distribution of amino acids at this site is given by

(2) $$\pi_{L,X}=\frac{\lambda_{X}e^{4N_{e}m_{L,X}}}{\sum_{{\text{X}}^{\prime }}\lambda_{{\text{X}}^{\prime }}e^{4N_{e}m_{L,X^{\prime }}}}$$

where $\lambda_{X}$ represents the results of the degeneracy of the genetic code and codon biases. Reversibility in the model requires that $v_{\text{XY}}\lambda_{X}=v_{\text{YX}}\lambda_{Y}=v_{\text{XY}}^{S}$ where $v_{\text{XY}}^{S}$ is symmetric ( $v_{\text{XY}}^{S}=v_{\text{YX}}^{S}$). Using these relationships, we can express the substitution rate in terms of equilibrium frequencies as

(3) $$Q_{L,\text{XY}}=v_{\text{XY}}\frac{\ln\left(\frac{\lambda_{X}\pi_{L,Y}}{\lambda_{Y}\pi_{L,X}}\right)}{1-\left(\frac{\lambda_{Y}\pi_{L,X}}{\lambda_{X}\pi_{L,Y}}\right)}$$

If we approximate $\ln(\lambda_{Y}\pi_{X})≃\lambda_{Y}\pi_{X}-1$, Eq. (3) reduces to $Q_{L,\text{XY}}≃v_{\text{XY}}^{S}\pi_{L,Y}$, an amino acid version of the Felsenstein 81 model (F81^29^), except that the equilibrium frequencies are specific for each site in the protein. Note that selective constraints are not otherwise present in this model—there is, for example, no exchangeability matrix encoding physicochemical similarities.

Using this approximation, we can generate a corresponding standard model by averaging fluxes and equilibrium distributions over sites, leading to

(4) $${\hat{Q}}_{\text{XY}}=\frac{{〈\pi_{L,X}Q_{L,\text{XY}}〉}_{L}}{{〈\pi_{L,X}〉}_{L}}=v_{\text{XY}}^{S}\left(1+\frac{{\text{Cov}(\pi }_{L,X}{,\pi }_{L,Y})}{{\hat{\pi }}_{Y}{\hat{\pi }}_{Y}}\right){\hat{\pi }}_{Y}$$

where ${\hat{\pi }}_{X}={〈\pi_{L,X}〉}_{L}$. The corresponding symmetric exchangeability matrix is given by

(5) $$S_{\text{XY}}=v_{\text{XY}}^{S}\left(1+\frac{{\text{Cov}(\pi }_{L,X}{,\pi }_{L,Y})}{{\hat{\pi }}_{Y}{\hat{\pi }}_{Y}}\right)$$

As can be seen by (4)) and (5), the substitution rate depends upon the covariances of the equilibrium frequencies, which represents the tendency of the old and new amino acids to be acceptable at the same sites.

**Figure 1:**
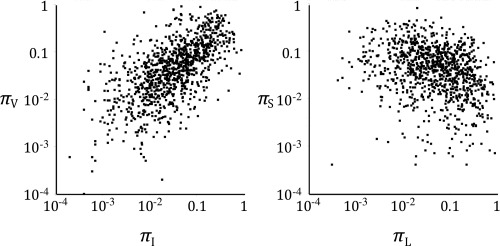
Correlation between the equilibrium frequencies of a random selection of sites from the Pfam database,^30^ for two similar amino acids [isoleucine (I) and valine (V), left] and two dissimilar amino acids [leucine (L) and serine (S), right].

Estimates for the covariances in the equilibrium frequencies can be obtained by considering sets of aligned homologous proteins. Figure 1 shows the estimated equilibrium frequencies for isoleucine and valine, and leucine and serine, at a random sample of sites from alignments in the Pfam 29.0 database.^30^ In these representative examples, as well as with other pairs, the correlations between the site‐specific equilibrium frequencies are highly indicative of the physicochemical similarities between the amino acids.

**Figure 2:**
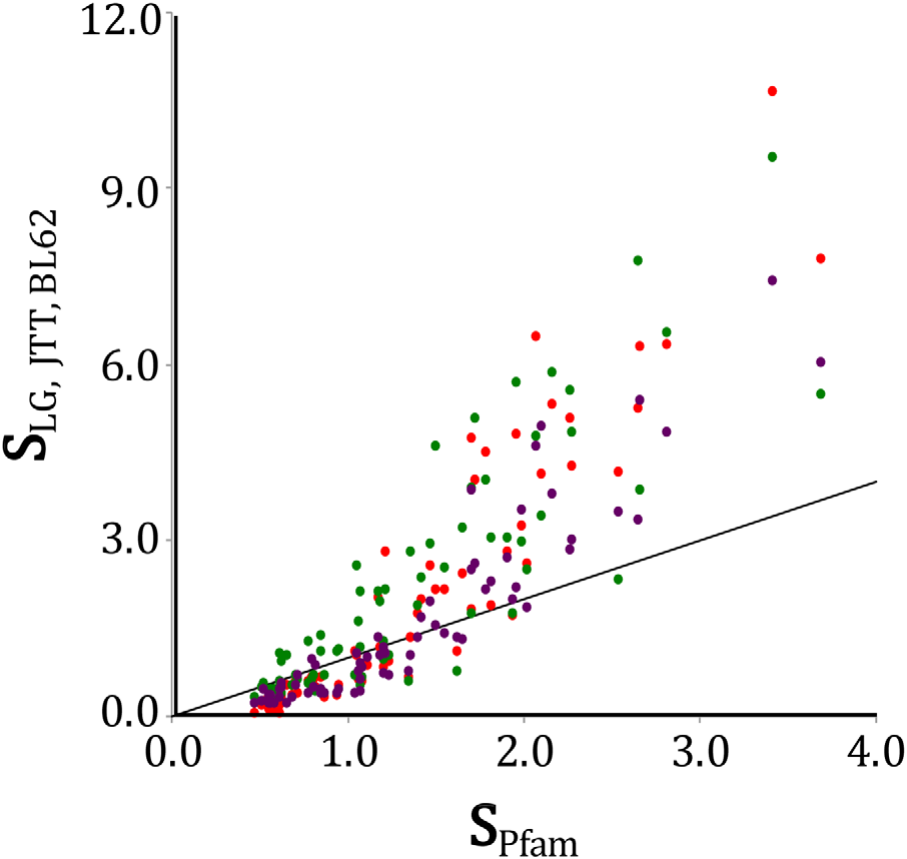
Predicted relative exchangeabilities (S) calculated from equilibrium amino acid frequencies and from a set of standard substitution matrices. The site‐specific amino acid frequencies were based on Pfam protein alignments and calculated with Eq. (5), whereas the substitution matrix exchangeabilities were calculated for Le and Gascuel^15^ (red), JTT^13^ (green), and Blossum62^12^ (purple). In all cases, the rate matrices from which the exchangeabilities were derived were normalized so that the average substitution rate was 1.0.

Empirical amino acid substitution models (e.g., WAG,^14^ JTT,^13^ LG,^15^ and Blossum62^12^) calculate the average substitution probabilities among amino acids over a large number of proteins and sites. By normalizing substitution matrices to one expected substitution per unit time, we can directly compare exchangeability parameters from these matrices to those from substitution matrices obtained by applying Eq. (5) to data extracted from the Pfam database (ignoring $v_{\text{XY}}^{S}$). Although these substitution matrices are obtained from quite different datasets, there is a strong and consistent correlation between estimates of exchangeabilities predicted by biophysical models and those estimated by more standard methods (Fig. 2). This highlights the connection between the correlations in equilibrium frequencies among a large variety of different sites in the biophysics based models, and the estimated exchangeabilities of standard substitution models where this variation is ignored. There is a discrepancy, however, in that the exchangeability values derived from the Pfam data have substantially faster relative rates for the slower substitutions compared with the empirical methods.

### Understanding the magnitude of substitution rates: the constraints at different sites

We can investigate the magnitude of substitution rates by considering the strength of selection acting on different positions. Individual sites in proteins have different constraints—sites on the exterior of the protein are often under weak selection and can accept most hydrophilic (and some hydrophobic) amino acids, while other sites are completely conserved over long evolutionary time scales, indicating that the site can only accept a single amino acid. We can quantify the degree of acceptable variation at different sites by calculating the sequence entropy, using ideas from information theory.^31^ By taking the exponent of the sequence entropy at any site *L* we arrive at the effective number of acceptable amino acids $\Omega_{L}$, equal to

(6) $$\Omega_{L}=\exp\left(-\sum_{X}\pi_{L,X}\ln\pi_{L,X}\right)$$

$\Omega_{L}$ is equal to the number of amino acids that would have the same sequence entropy if all of these amino acids were equally likely.

**Figure 3:**
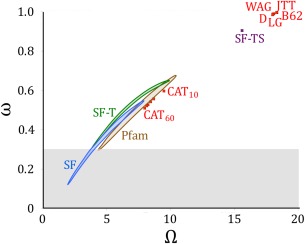
Relationship between effective number of permissible amino acids and the corresponding amino acid substitution rate compared to neutral evolution, as calculated with Equation 3. Standard substitution models (red: WAG,^14^ JTT,^13^ LG,^15^ Dayhoff (D),^11^ Blossum62 (B62)^12^) average over the selective constraints at many sites, resulting in a large number of permissible amino acids corresponding to a near‐neutral rate of evolution, far from that observed in biological proteins (shaded region).^32^ Including spatial heterogeneity as in the CAT models (red: C10, C20, C30, C40, C50, C60)^36^ significantly restricts the acceptable amino acids, as does computing equilibrium frequencies based on Pfam protein alignments (brown),^30^ but still cannot generate reasonable substitution rates. Simulations that include temporal heterogeneity (Stokes Fisher model, SF, blue)^4^, ^5^, ^10^, ^42^ achieve substitution rates close to biological proteins without ad‐hoc rate scaling factors, despite omitting selective constraints on function. Averaging the Stokes Fisher simulation results to remove temporal heterogeneity (SF‐T) or both spatial and temporal heterogeneity (SF‐TS) yields results similar to other approaches that ignore such heterogeneities.

We can now ask, what is the relationship between the strength of the selective constraints, represented by the range of permissible amino acids, and the expected and observed substitution rates in different models. For standard substitution models, every site has the same set of equilibrium frequencies and thus the same effective number of acceptable amino acids. Because every amino acid is observed in a reasonable fraction of sites, $\Omega_{L}$ is approximately 18, corresponding to few selective constraints. We can now ask, from a biophysical perspective, what substitution rates would we expect to observe in sites with this value of $\Omega_{L}$? Calculating the substitution rate for all pairs of amino acids based on the Kimura formula [Eq. (3)], one would expect a nonsynonymous rate (relative to the neutral rate) in excess of 0.95 (Fig. 3), far above what is commonly observed in proteins.^32^

According to our biophysical understanding, selective constraints determine which amino acids are acceptable in each location, represented by shifts in equilibrium frequencies, which have an impact on the probabilities of different mutations being accepted. Stronger selective constraints manifest themselves as fewer acceptable amino acids and correspondingly slower substitution rates. General empirical models, however, represent the equilibrium distribution at all sites with the same broad range of acceptable amino acids, precluding the representation of selection in a biologically coherent manner: it is difficult to imagine a form of selection acting on a various locations in a protein that would reduce the substitution rate at some sites while leaving the equilibrium distribution of amino acids unchanged. Instead, these models allow for the effects of selective pressure on substitution rates using *ad hoc* multipliers. The most common approach, used by the general substitution matrices considered above, is to simply scale branch lengths by amino acid substitutions decoupled from neutral nucleotide substitution rates. Alternatively, average and site‐specific deviations from neutral expectation may be accommodated in standard codon models^6^, ^7^, ^33^ using a set of values for the ratio of nonsynonymous to synonymous substitution rates that vary among sites.

The simplicity and computational tractability of these empirical approaches has allowed their widespread use in a range of different applications, but is achieved at the expense of adding terms without a clear biological basis. These approaches reduce our ability to interpret the results of these models directly in terms of the underlying biophysics and evolutionary biology. In particular, investigations into the nature and cause of varying selective constraint among sites are hampered by using models that deny such variation.

One approach to resolving these issues is to include spatial heterogeneity in the substitution models. By encoding the selective constraints specific to each site, these constraints can be made more specific, and therefore more restrictive, reducing the effective number of acceptable amino acids at each site and reducing the expected substitution rate relative to the neutral substitution rate in a more biologically reasonable manner. Two ways in which this is currently achieved are by estimating site‐specific equilibrium frequencies,^8^, ^23^ or by creating a mixture model that allows different substitution processes among classes.^34^, ^35^, ^36^ As an example of the former approach, we used Pfam sets of aligned sequences^30^ to define site‐specific equilibrium frequencies, and then used these frequencies to calculate the average effective number of acceptable amino acids, as well as the expected average substitution rate. As an example of the latter approach, Quang and colleagues developed sets of ‘CAT’ substitution models in which the substitution process is defined by equilibrium amino acid frequencies and the fraction of all sites that would be expected to be a member of each class.^36^ Both of these approaches of including site heterogeneity result in a significantly lower number of acceptable amino acids and thus a lower expected substitution rate than the general models (Fig. 3). However, the effective numbers of acceptable amino acids are still large enough that the predicted substitution rates compared to neutral expectation are substantially higher than observed in biological proteins.

It appears that to reduce the expected substitution rate to levels compatible with observation without the introduction of *ad hoc* multipliers, the number of permissible amino acids must be less than predicted by these spatial heterogeneity methods. One possible explanation is temporal heterogeneity, which we turn to next. Temporal heterogeneity arises naturally from epistasis or coevolution, in which changes in the protein modify evolutionary dynamics at interacting sites. Kondrashov and colleagues previously argued that pervasive epistasis was required to explain observed substitution rates,^37^ although Plotkin and colleagues demonstrated that the analysis was fundamentally flawed and insufficient to prove epistasis.^38^ The evidence for epistasis or coevolution based on convergence data^10^ and phylogeny‐based coevolutionary or correlated mutation analyses^9^, ^39^, ^40^, ^41^ is more direct and in our opinion more convincing.

### Time heterogeneity of substitution rates

**Figure 4:**
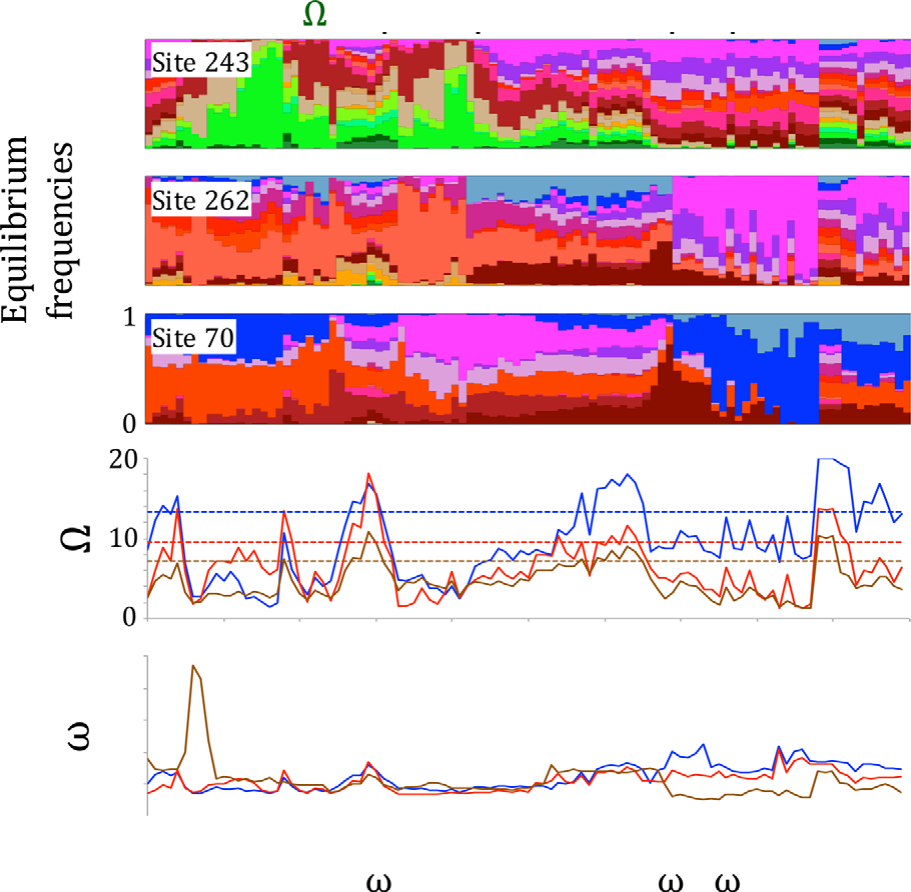
Fluctuating equilibrium frequencies, permissible amino acids and rates over time in SF simulations. (A) Equilibrium frequencies of the amino acids at site three different sites (243, exposed; 262, partially buried; 70 buried) during evolutionary simulation. The simultaneous changes of propensities at different sites reflect the nature of a protein as an integrated entity. (B) Effective number of permissible amino acids (Ω), including instantaneous values (solid), and calculated using average equilibrium distributions (dashed), for sites 243 (blue), 262 (red), and 70 (brown). (C) Instantaneous substitution rates (ω) at sites 243, 262, and 70, over the same interval.

In earlier work, we performed long computational simulations of the evolution of a 300‐residue purple acid phosphatase under selection for thermostability.^4^, ^5^, ^10^, ^42^ We will refer to these as Stokes–Fisher simulations, or SF. We noted that the equilibrium frequencies of the various amino acids varied over time, as shown for three different sites in Figure 4. Not only does the corresponding effective number of available amino acids vary during the evolutionary period, but the values are generally substantially smaller than those calculated ignoring temporal heterogeneity, and result in substitution rates more similar to those observed (see blue SF versus brown pfam 68% credible regions in Fig. 3).

We next considered what would happen to this data if temporal and/or spatial heterogeneity were ignored. When we averaged the SF data over temporal heterogeneity, recovered values of $\Omega_{L}$ and expected substitution rates were similar to that of the site‐specific CAT‐60 model (green SF‐T 68% credible region, Fig. 3). When we averaged over both temporal and spatial heterogeneity the results were just slightly lower than the general substitution models (purple SF‐TS point, Fig. 3). Thus, the effective number of amino acids and substitution rates compared to neutral expectation predicted by current general and site‐specific substitution models are consistent with our expectations from epistatic fluctuations generated by thermodynamically stable evolutionary simulations. This suggests that to obtain a more extensive mechanistic explanation for molecular evolution, we should include temporal, as well as spatial, heterogeneity.

## Conclusions

In this paper, we show that the presence of spatial and temporal heterogeneity can be used to link biophysical models of proteins with standard substitution models. According to biophysical models, if a nonconservative substitution from *X* to *Y* is deleterious at a particular point in time, and therefore slow, the reverse substitution from *Y* to *X* should be advantageous and therefore fast. Yet when averaged over time and sites, the estimated rate for both substitutions should be slow. This seeming paradox can be resolved by considering the number of such substitutions observed. A substitution requires an initial amino acid, generally with a high propensity for that site (or else it would not be present there) and a new amino acid, also with high propensity for that site (or else this substitution is likely highly deleterious and unlikely to occur). The number of observed substitutions therefore depends on the number of sites where both amino acids are acceptable, that is, on the correlations between the propensities of the amino acids at different sites in the protein, as expressed in Eq. (4). As this correlation is higher for amino acids with similar physicochemical properties, we expect that the average substitution rate for similar amino acids is higher than for dissimilar amino acids, as observed in standard models. Even though standard models misrepresent the rates at any specific site, seemingly indicating that nonconservative substitutions in either direction are deleterious, they can capture the manner in which the average flux relates to the average equilibrium frequencies. In this way, the heterogeneity ignored by standard substitution models is actually *responsible* for the resulting form of these models, and their tendency to favour conservative substitutions.

As demonstrated with the CAT mixture models as well as the models based on Pfam emission probabilities, spatial heterogeneity alone is likely inadequate to explain the low number of amino acids acceptable at any site and time, and it is difficult to obtain substitution rates as slow as observed without incorporating empirical rate factors that lack biological meaning. This highlights the importance of understanding temporal heterogeneity in any biologically based model of substitution rates. The importance and even existence of epistatic interactions has sometimes been controversial, although experimental observations indicate that only the magnitude of the effects are debatable;^5^, ^43^, ^44^, ^45^, ^46^, ^47^ such interactions are, as would be expected, relatively small when considering closely‐related viral strains, and larger when more substantial evolutionary distances are involved. Interestingly, recent work suggests that such epistatic interactions are enhanced in naturally occurring substitutions compared with random mutations.^47^ The effect is also magnified during adaptive functional changes, as the protein has to adjust to more nonconservative substitutions.

It is doubtful that the equilibrium frequencies estimated from the Pfam alignments accurately represent the true equilibrium amino acid frequencies. Firstly, according to the perspective presented here, the equilibrium acid frequencies are themselves time‐dependent, and thus cannot be uniquely defined over a finite time period. Rather, models such as Pfam represent the equilibrium amino acid frequencies averaged over the evolutionary time period covered by a sequence alignment. Moreover, the equilibrium frequencies are based on a finite set of examples, and will be influenced by the biases present in current sequence databases. The examples are also not independent examples, but are phylogenetically related; sequence‐weighting methods for dealing with these dependencies remain imperfect, and the estimates would potentially be biased even if the equilibrium amino acid frequencies were not fluctuating over time. The similarity in the results of the analysis of Pfam sequence alignments, the CAT models incorporating site heterogeneity, and the time‐averaged SF models shown in Figure 3 suggest that the Pfam models are not grossly inadequate for our purposes.

Although it is our belief that time and spatial heterogeneity in substitution rates need to be better understood and incorporated in the models of the future, there are two major challenges that remain. Firstly, the computational challenges are substantial, and classical methods of performing likelihood calculations on trees are not adaptable to the challenge. We and others have been using next generation methods Bayesian methods that augment substitutions where they most plausibly occurred on the tree, updating the augmented substitutions with successive Markov chain iterations (e.g., Refs.^48^, ^49^, ^50^) Such methods can be made to scale well with large numbers of models and states, particularly with partial sampling of substitution histories and uniformization.^50^, ^51^ The second major challenge is to understand how temporal and spatial variability should be modelled and parameter space simplified given an almost infinite number of possibilities. Simple rate‐switching models such as covarion and related models^52^, ^53^ accommodate the effect of fluctuating constraints on fluctuating rates, and may therefore in some cases be useful, but they do not reflect the fluctuating constraints themselves. It is our view that such models should be carefully defined based on biological mechanistic considerations, and that much of future work should focus on what these mechanisms are and what is detectable given the limitations of sequence acquisition from extant organisms.

## Methods

### Covariation between equilibrium frequencies

For the covariation between equilibrium frequencies shown in Figure 1, random sites were selected from the Pfam 29.0 database^30^ set of protein alignments. For each of these states, the equilibrium frequencies for each pair of amino acids was extracted employing sequence weighting developed by Henikoff and Henikoff.^54^ Only alignments larger than 100 sites with more than 100 sequences were considered, and sites with gaps in over 50% of the (weighted) sequences were excluded. These equilibrium frequencies were used to calculate exchangeabilities (Fig. 2) using Eq. (5).

### Effective number of accessible amino acids and corresponding substitution rate

For standard substitution models (e.g., Dayhoff, WAG, JTT, Blossum62, LG), published equilibrium frequencies were used to calculate the effective number of accessible amino acids using Eq. (6). The overall substitution rate relative to the neutral rate ω was also calculated using these equilibrium frequencies using

(7) $$\omega =\frac{\sum_{〈\text{XY}〉}\pi_{X}Q_{\text{XY}}}{\sum_{〈\text{XY}〉}\frac{d_{X}}{61}v_{\text{XY}}^{S}},$$

where $Q_{\text{XY}}$ is calculated with Eq. (3) using the number of codons encoding each amino acid (*d*_X_) to calculate $\lambda_{X}$, and $v_{\text{XY}}^{S}$ is computed based on the K80 nucleotide model ( K=2).^55^ Calculations with the CAT models^36^ were performed in a similar manner, averaging over the set of site class models. Each point in the Pfam set^30^ represents the average over a single protein alignment.

### Evolutionary simulations

The evolutionary simulations have been described previously.^4^, ^10^, ^42^ Briefly, we simulated the evolution of a 300‐amino acid phosphatase (PDB 1QHW^56^). The free energy of a sequence in this structure was estimated based on the sum of pair‐wise contact energies using contact potentials estimated by Miyazawa and Jernigan.^57^ We also calculated the free energy for an ensemble of 55 alternative folds, and these calculations were used to estimate the free energy for a much larger ensemble (10^160^) of unfolded structures. These calculations allowed us to calculate the free energy of folding Δ*G*([*A*_i_]) for any amino acid sequence [*A*_i_]. The fitness of a sequence was then calculated as the probability of being folded at equilibrium

(8) $$f(\{A_{i}\})=\frac{\exp\left(-\frac{\Delta G(\{A_{i}\})}{kT}\right)}{1+\exp\left(-\frac{\Delta G(\{A_{i}\})}{kT}\right)},$$

where *kT* is the product of the Boltzmann constant and the temperature.

Starting out with a random sequence of codons (excluding stop codons), the rate of all possible single‐base substitutions was calculated using Eq. 2, using the K80 nucleotide model ( K=2).^55^ Time was advanced by an amount chosen from an exponential distribution based on the sum of the substitution rates, while a substitution was accepted proportional to the relative rates. Simulations were allowed to reach a state of mutational drift–selection balance, in which the selective pressure for increased stability was in balance with the much larger number of destabilising mutations. After this point, all results were obtained with simulations in which the fitness of the protein underwent random unbiased fluctuations. The simulations were completely transparent, meaning that the timing and nature of every substitution, the instantaneous substitution rates, and the instantaneous equilibrium frequencies at every location were accessible.
